# PDH Inhibition in *Drosophila* Ameliorates Sensory Dysfunction Induced by Vincristine Treatment in the Chemotherapy-Induced Peripheral Neuropathy Models

**DOI:** 10.3390/biomedicines13040783

**Published:** 2025-03-24

**Authors:** Harim Song, Sohee Kim, Ji Eun Han, Kyong-hwa Kang, Hyongjong Koh

**Affiliations:** 1 Department of Pharmacology, Dong-A University College of Medicine, Busan 49201, Republic of Korea; sere805163@naver.com (H.S.); thgml193@naver.com (S.K.); zbwldms12@naver.com (J.E.H.); 2 Department of Translational Biomedical Sciences, Dong-A University College of Medicine, Busan 49201, Republic of Korea; 3 Neuroscience Translational Research Solution Center, Dong-A University College of Medicine, Busan 49201, Republic of Korea

**Keywords:** chemotherapy-induced peripheral neuropathy (CIPN), vincristine, *Drosophila*, thermal nociception, pyruvate dehydrogenase (PDH)

## Abstract

**Background/Objectives:** Chemotherapy-induced peripheral neuropathy (CIPN) is a significant dose-limiting side effect of many effective anticancer agents, including vincristine. While CIPN adversely affects both oncological outcomes and the quality of life for cancer patients, the in vivo mechanisms behind CIPN pathology remain largely unknown, and effective treatments have yet to be developed. In this study, we established a novel *Drosophila* model of CIPN using vincristine to explore the molecular mechanisms underlying this condition. **Methods:** We assessed the impact of vincristine exposure on thermal nociception in *Drosophila* larvae using a programmable heat probe. Additionally, we investigated vincristine-induced mitochondrial dysfunction and dendritic abnormalities in class IV dendritic arborization (C4da) neurons with various fluorescent protein markers. **Results:** We found a dose-dependent increase in thermal hypersensitivity, accompanied by changes in the sensory dendrites of C4da neurons in vincristine-treated fly larvae. Moreover, vincristine significantly enhanced mitochondrial ROS production and mitophagy—a selective autophagy that targets dysfunctional mitochondria—indicating vincristine-induced mitochondrial dysfunction within C4da neurons. Surprisingly, inhibiting the pyruvate dehydrogenase complex (PDH), a key mitochondrial metabolic enzyme complex, effectively rescued the mitochondrial and sensory abnormalities caused by vincristine. **Conclusions:** Findings from this first *Drosophila* model of vincristine-induced peripheral neuropathy (VIPN) suggest that mitochondrial dysfunction plays a critical role in VIPN pathology, representing PDH as a potential target for the treatment of VIPN.

## 1. Introduction

Chemotherapy-induced peripheral neuropathy (CIPN) is a significant neurotoxic side effect associated with many effective anticancer agents. A meta-analysis of clinical studies involving over 4000 patients found that CIPN occurs in 68% of patients within the first month following chemotherapy [1]. Common symptoms of CIPN include sensory nerve abnormalities such as numbness, paresthesia, allodynia, and hyperalgesia [2]. These painful adverse effects can lead to treatment delays or even discontinuation, which may result in poor oncological outcomes [1]. Additionally, more than 30% of patients experience chronic sensory symptoms that can last for several days or even years, adversely affecting the quality of life for cancer patients and survivors [1].

Vincristine, a microtubule-targeting agent, is widely used to treat leukemia, lymphomas, brain tumors, and solid tumors in both adults and children [3]. It binds to tubulin and inhibits its polymerization into microtubules, thereby preventing the formation of the mitotic spindle and leading to cell cycle arrest and apoptosis [4]. As neurons depend on microtubules to maintain their unique structure and function, disrupted microtubule dynamics are thought to be the source of vincristine’s neurotoxicity [5]. Vincristine primarily impacts the peripheral nerves, resulting in sensory symptoms such as numbness, pain, and tingling, as well as motor symptoms, including muscle weakness and reduced reflexes [6,7]. Additionally, vincristine can cause autonomic dysfunction, resulting in urinary retention, constipation, and orthostatic hypotension [8,9]. Because vincristine inhibits microtubule-based axonal transport and activates the axon degeneration program, researchers have focused on axon degeneration pathways in various cell or rodent vincristine-induced peripheral neuropathy (VIPN) models to develop new therapeutics [5]. However, the molecular mechanisms underlying VIPN pathology remain largely unknown, and effective therapeutics are not yet available.

*Drosophila* models have elucidated the molecular mechanisms underlying various physiological functions, including pain nociception [10]. When exposed to noxious thermal heat, third-instar larvae display characteristic rolling behavior, which has been utilized to identify conserved genes essential for nociception [10]. This response stems from the activation of class IV dendritic arborization (C4da) sensory neurons at the site of stimulation [11]. In previous *Drosophila* studies, the genotoxic effects of vincristine were tested in some somatic cells [12,13], yet its neurotoxic impact has not been explored in *Drosophila* models.

In this study, we examined the effect of vincristine exposure on thermal nociception in *Drosophila* larvae and found a significant dose-dependent increase in pain perception, which correlated with changes in the sensory dendrites of C4da neurons. Further analysis showed that vincristine elevated mitochondrial reactive oxygen species (mt-ROS) production and mitophagy, indicating mitochondrial damage in C4da neurons caused by vincristine. Importantly, inhibiting the pyruvate dehydrogenase complex (PDH), a crucial mitochondrial metabolic enzyme complex that produces Acetyl-Coenzyme A (Acetyl-CoA) and the byproduct hydrogen peroxide (H_2_O_2_) [14], markedly reduced mt-ROS and mitophagy in vincristine-treated C4da neurons. Moreover, PDH inhibition alleviated thermal hyperalgesia and sensory dendrite alterations induced by vincristine treatment. These findings suggest that regulating mitochondrial metabolism may serve as a novel therapeutic strategy for vincristine-induced peripheral neuropathy (VIPN).

## 2. Materials and Methods

### 2.1. Drosophila Strains

The *w^1118^* (3605), *elav-GAL4* (458), *ppk-GAL4* (32078), and *GFP RNAi* (9331) lines were obtained from the Bloomington Stock Center (Bloomington, IN, USA). The *Pdha1 RNAi* line (v107209) was purchased from the Vienna Drosophila Resource Center (Vienna, Austria). The *UAS-PDK*, *UAS-mt-Keima*, and *PDP^P^* (*PDP^G1628^*) fly lines were generated as described previously [15,16]. The *UAS-CD4-tdTomato* (*CD4-tdTom*) and *ppk1a-GAL4* lines were kindly provided by Dr. Y.N. Jan. The *UAS-mito-roGFP-orp1* line was a gift from Dr. T.P. Dick. All fly stocks were fed a standard diet consisting of 5% yeast, 3.8% cornmeal, 7% dextrose, 0.5% agar, 0.45% propionic acid, and 0.75% tegosept in ethanol and were housed at a normal temperature of 25 °C with humidity levels maintained at 60%.

### 2.2. Drug Treatments

Drugs were administered following the feeding regimen. In summary, 20 virgin female flies mated with 50 male flies for 48 to 72 h, and embryos were collected for 2 to 4 h. The embryos were grown for 72 h into 3rd-instar larvae. The larvae were rinsed with deionized water and fed standard fly media containing 30 μM or 100 μM vincristine without gavage for 48 h.

### 2.3. Larval Thermal Nociception Assays

The larval thermal nociception assays were performed to analyze the vincristine-induced heat hypersensitivity phenotypes. The 3rd-instar larvae (120 h after egg laying (AEL)) were rinsed with deionized water and placed gently on a petri dish. After acclimation for 10 s, the larval abdominal segments A4–A5 were microscopically touched with a custom 0.6 mm wide heat probe (40 °C) controlled with a microprocessor. The time taken to induce rolling reactions such as aversive corkscrews was measured as a withdrawal delay with a 20 s cutoff. Larvae that did not exhibit a rolling response within 20 s were considered unresponsive. At least 50 larvae were analyzed for each thermal nociceptive assay, and the results are presented as the mean values with SD.

### 2.4. Analysis of C4da Neuron Dendrites

To analyze the dendritic structure of C4da neurons in the abdominal segment A4 of the 3rd-instar larvae, images of the fluorescent plasma membrane marker CD4-tdTomato were obtained using an LSM 800 confocal microscope (Zeiss, Oberkochen, Germany) at the Neuroscience Translational Research Solution Center (Busan, Republic of Korea). Confocal image stacks of the larval C4da neuron dendrites were converted to maximum intensity projections using Zeiss Zen software (Ver 3.4). The dendritic length and dendritic branching number were analyzed in ImageJ software by using the skeleton plugin function (Ver 1.53t, NIH, Bethesda, MD, USA). At least 15 larvae per genotype were analyzed. All of the results are presented as the mean value with SD.

### 2.5. Measurement of Mitochondrial ROS (mtROS) of C4da Neurons

For mtROS imaging in C4da neurons, we examined 3rd-instar larvae expressing mito-roGFP2-Orp1 using a Zeiss LSM 800 confocal microscope with either a 405 nm (oxidized) or a 488 nm (reduced) excitation laser, capturing emissions at 520 nm. For each group, we imaged 15 larvae and measured the 405 nm/488 nm fluorescence intensities using Zeiss Zen software. The results are presented as mean values with SD.

### 2.6. Measurement of Mitophagy Levels

Mitophagy levels were quantified using mt-Keima, the mitochondrial-targeted fluorescent protein described previously [15]. To assess the level of mitophagy in C4da neurons, 3rd-instar larvae expressing mt-Keima were examined with a Zeiss LSM 800 confocal microscope. Mt-Keima fluorescence was analyzed, and the subsequent mitophagy level (% mitophagy) was calculated using the Zeiss Zen software as previously described [16]. The average mitophagy level (±SD) was obtained from ten independent samples.

### 2.7. Quantitative RT-PCR

After eclosion, 20 male flies were incubated for 2 days on standard fly food. Total RNA was isolated from 20 fly heads and reverse transcribed as previously described [15]. Quantitative real-time RT-PCR was performed using SYBR Premix Ex Taq II (Takara, Japan) on the QuantStudio 3 system (Thermo Fisher Scientific, Waltham, MA, USA), with rp49 levels serving as an internal control. The results are presented as fold changes relative to the control. Average mRNA levels ± SD were calculated from three independent experiments. The following primer pairs were used: rp49 F (GCT TCA AGA TGA CCA TCC GCC C), R (GGT GCG CTT GTT CGA TCC GTA AC), Pdha1 F (GTG TCC ACG GAT GGA CCT AC), and R (CCA TGT TGT AAG CCT CGA ACA C).

### 2.8. Measurement of PDH Activity

Ten 3rd-instar larvae were homogenized in 100 µL of PDH assay buffer from the pyruvate dehydrogenase activity assay kit (Sigma Aldrich, St. Louis, MO, USA, #MAK183). PDH activity was assessed by monitoring the kinetics of NADH production at 450 nm at 37 °C using a SpectraMax ID3 multi-mode microplate reader (Molecular Devices, San Jose, CA, USA). The average relative PDH activity with ±SD was calculated from three independent experiments.

### 2.9. Statistical Analyses

A one-way analysis of variance with Sidak correction was used to compare three or more groups. We used a two-tailed Student’s *t*-test to compare the two groups. Statistical significance was set at *p* < 0.05.

### 2.10. Genotypes

**Figure 1:**
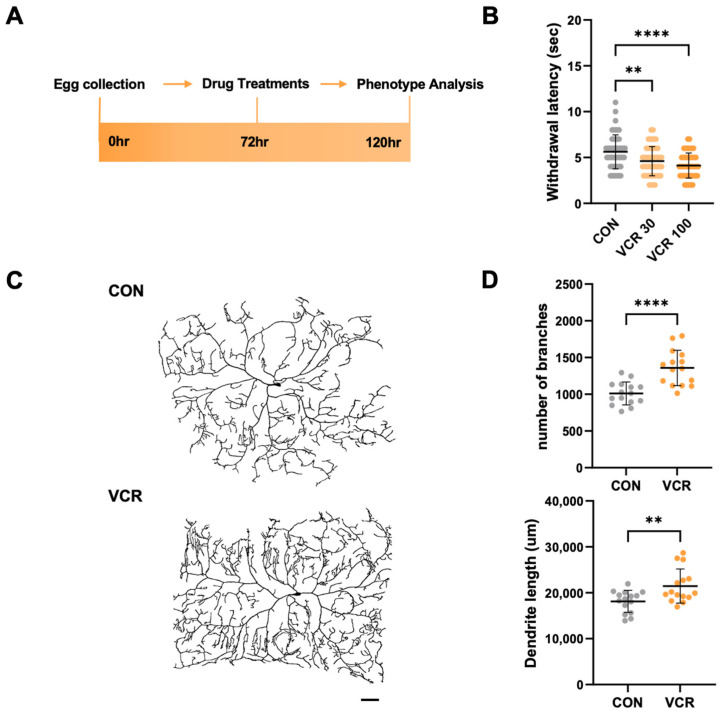
Vincristine-induced thermal hypersensitive phenotypes in *Drosophila* larvae. (**A**) Experimental design for vincristine treatment and the thermal nociception assay. The early third-instar larvae were transferred to media containing DMSO vehicle or vincristine (0, 30, 100 μM) at 72 h AEL. The larvae were treated for 48 h, and the thermal nociception response was observed at 120 h AEL. (**B**) Thermal nociceptive withdrawal due to 40 °C stimulation was assessed after treatment with vehicle (CON), 30 μM vincristine (VCR 30), or 100 μM vincristine (VCR 100), as in (**A**) (n = 50 per group). (**C**) Representative images of C4da neurons in abdominal segment A4 of third-instar larvae expressing the plasma membrane marker CD4-tdTom after 48 h of exposure to either vehicle (CON) or 100 μM vincristine (VCR). Scale bars indicate 50 μm. (**D**) Quantification of the dendrite length and the number of dendritic branch points of C4da neurons (n = 15 per group). Significance was determined using one-way ANOVA with Sidak correction (**, *p* < 0.01; ****, *p* < 0.0001). Error bars represent mean ± SD.

**Figure 2:**
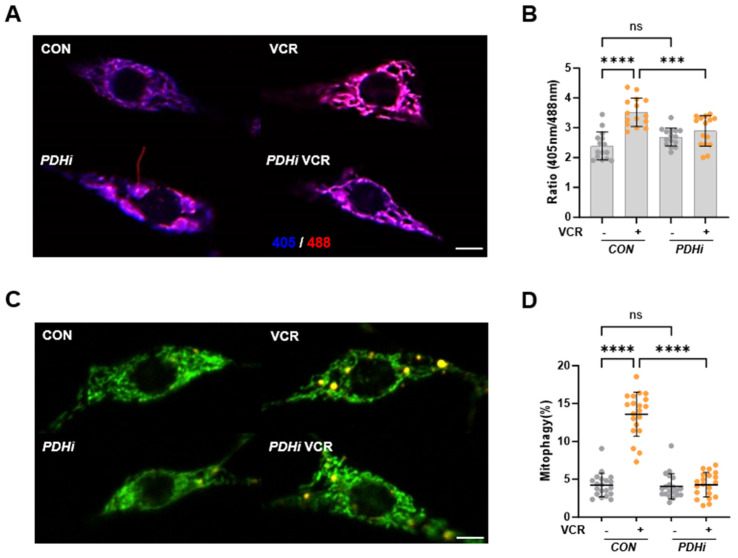
Vincristine increased mitochondrial ROS and mitophagy levels in C4da neurons. (**A**) Representative mito-roGFP2-Orp1 fluorescence images of C4da sensory neurons in control (*CON*) and *Pdha1* RNAi-expressing (*PDHi*) larvae treated with 100 μM vincristine (VCR) for 48 h. Scale bars, 5 μm. (**B**) Quantitative analysis of the mitochondrial ROS levels of the C4da sensory neurons (n = 15 per group). (**C**) Representative mt-Keima fluorescence images of C4da sensory neurons in control (*CON*) and *Pdha1* RNAi-expressing (*PDHi*) larvae treated with 100 μM vincristine (VCR) for 48 h. Scale bars, 5 μm. (**D**) Quantitative analysis of the mitophagy of C4da sensory neurons (n = 20 per group). Significance was determined using one-way ANOVA with Sidak correction (*** *p* < 0.001; **** *p* < 0.0001; ns, not significant). Error bars indicate mean ± SD.

**Figure 3:**
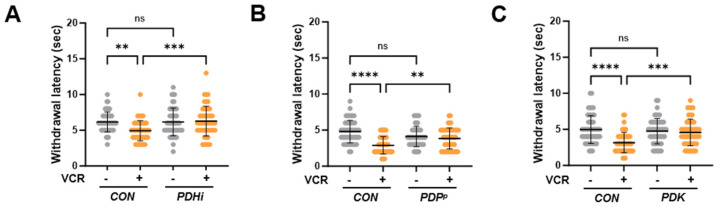
PDH inhibition mitigated vincristine-induced sensory defects. Thermal nociceptive withdrawal in response to 40 °C stimulation was evaluated after 100 μM vincristine treatment (VCR). (**A**) Mean withdrawal latency for control (*CON*) and *Pdha1* RNAi-expressing (*PDHi*) larvae (n = 50 per group). (**B**) Mean withdrawal latency for control (*CON*) and *PDP* mutant (*PDP^P^*) larvae (n = 50 per group). (**C**) Mean withdrawal latency for control (*CON*) and PDK-expressing (*PDK*) larvae (n = 50 per group). Significance was assessed using one-way ANOVA with Sidak correction (** *p* < 0.01; *** *p* < 0.001; **** *p* < 0.0001; ns, not significant). Error bars represent mean ± SD.

**Figure 4:**
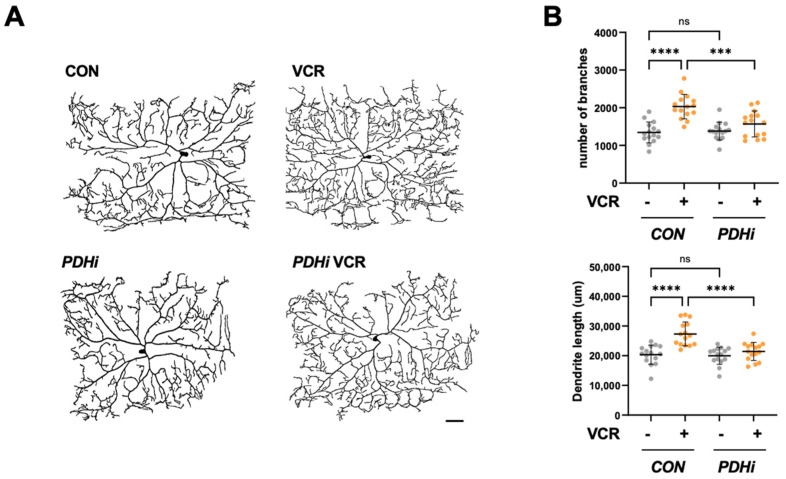
PDH inhibition alleviated vincristine-induced sensory dendritic defects. (**A**) Representative images of C4da neurons in abdominal segment A4 from control (*CON*) and *Pdha1* RNAi expressing (*PDHi*) larvae following 100 μM vincristine treatment (VCR). Scale bars, 50 μm. (**B**) Quantification of dendrite length and the number of dendritic branch points in C4da neurons (n = 15 per group). Significance was assessed using one-way ANOVA with Sidak correction (*** *p* < 0.001; **** *p* < 0.0001; ns, not significant). Error bars indicate mean ± SD.

Figure 1B: ppk-GAL4/UAS-RFP RNAi, Figure 1C,D: ppk1a-GAL4, UAS-CD4-tdTomato/+, Figure 2A,B: CON (ppk-GAL4, UAS-mito-roGFP2-Orp1/UAS-RFP RNAi); PDHi (ppk-GAL4, UAS-mito-roGFP2-Orp1/UAS-Pdha1 RNAi), Figure 2C,D: CON (UAS-GFP RNAi/+; ppk1a-GAL4, UAS-mt-Keima/+); PDHi (UAS-Pdha1 RNAi/+; ppk1a-GAL4, UAS-mt-Keima/+), Figure 3A: CON (ppk-GAL4/UAS-RFP RNAi); PDHi (ppk-GAL4/UAS-Pdha1 RNAi), Figure 3B: CON (w^1118^); PDP^P^ (PDP^P^/PDP^P^), Figure 3C: CON (ppk-GAL4/+); PDK (ppk-GAL4/UAS-PDK), Figure 4A,B: CON (UAS-GFP RNAi/+; ppk1a-GAL4, UAS-CD4-tdTomato/+); PDHi (UAS-Pdha1 RNAi/+; ppk1a-GAL4, UAS-CD4-tdTomato/+), Figure S1: CON (elav-GAL4/+; UAS-RFP RNAi/+); PDHi (elav-GAL4/+; UAS-Pdha1 RNAi/+), Figure S2: ppk-GAL4/UAS-RFP RNAi.

## 3. Results

### 3.1. Vincristine Feeding to Drosophila Larvae Induces a Heat-Sensitive Phenotype with Alterations in Dendrites

To establish a fly VIPN model, we treated third-instar larvae (about 72 h AEL) with different doses of vincristine for 48 h (Figure 1A). We then assessed their sensory phenotypes using a recently developed *Drosophila* thermal nociception assay [17]. A microprocessor-controlled probe set to 40 °C, the temperature that elicited the most consistent thermal nociception responses in our previous experiments [17], was used to touch the larvae’s abdominal segments (A4–A5). We measured the withdrawal latency, which is the time required to trigger the characteristic corkscrew-like rolling response to the noxious heat. In this assay, control larvae treated with a vehicle exhibited a mean withdrawal latency (MWL) of 5.63 ± 1.84 s (Figure 1B). Based on previous *Drosophila* neuropathy models treated with 30 μM paclitaxel, another microtubule inhibitor that induces CIPN [18,19], we treated larvae with 30 μM vincristine. After the treatment, the larvae displayed a significantly reduced MWL (to 4.60 ± 1.57 s), showing that this vincristine dose is enough to elicit significant hyperalgesia in fly larvae (Figure 1B). Furthermore, when the vincristine dose was increased to 100 μM, a concentration previously noted to inhibit the growth of *Drosophila* tumor stem cells [20], the MWL was further diminished (Figure 1B). This dose-dependent hyperalgesia clearly shows that fly larvae are vulnerable to vincristine-induced thermal hypersensitivity.

To evaluate the effects of vincristine on larval C4da neurons, we genetically labeled the neurons with the plasma membrane marker CD4-tdTomato, enabling visualization of their terminal dendrite branches [21]. After 48 h of exposure to vincristine, following the heat probe assay regimen, we noted a significant increase in dendrite branch points and length in the C4da neuron located in the A4 abdominal segment, which is responsible for sensing thermal stimuli during the thermal nociception assay (Figure 1C,D). Interestingly, prior studies have indicated that paclitaxel also induced thermal hypersensitivity in *Drosophila* larvae while promoting increases in dendrite length and branching in C4da neurons [17,19]. These consistent findings demonstrate that inhibiting microtubule dynamics can result in a peripheral neuropathy phenotype in *Drosophila* larvae, suggesting that our vincristine feeding regimen serves as a reliable model for studying VIPN in *Drosophila*.

### 3.2. Vincristine Causes Mitochondrial Damage in C4da Neurons, Which Is Rescued by PDH Inhibition

Recent studies have highlighted the intrinsic role of mitochondrial dysfunction in the pathophysiology of VIPN. For instance, fluocinolone, a mitochondrial trafficking regulator, restores stalled anterograde axonal mitochondrial transport and mitigates axon degeneration during vincristine treatment. Moreover, mitoquinone (MitoQ), a mitochondrial-targeted antioxidant, significantly alleviated vincristine-induced pain sensitivity in mice [22]. Further analysis revealed that vincristine caused severe oxidative stress in the spinal cord of mice, which was countered by MitoQ treatment [22]. To investigate mitochondrial dysfunction following vincristine treatment, we assessed mitochondrial ROS levels, which indicate mitochondrial stress, in C4da neurons by expressing the in vivo mitochondrial H_2_O_2_ probe mito-roGFP2-ORP1 [23]. After vincristine exposure, the mitochondrial ROS level increased significantly in C4da neurons (Figure 2A,B), suggesting that vincristine induces mitochondrial damage in C4da sensory neurons.

Mitochondrial damage induces mitophagy, a selective autophagy that degrades dysfunctional or damaged mitochondria [24]. We also observed that mitochondrial damaging insults such as hypoxia and rotenone treatment induce mitophagy in *Drosophila* larvae [16]. Therefore, we next assessed the mitophagy level in C4da neurons using a pH-dependent fluorescent probe, mitochondria-targeted Keima (mt-Keima). In the cytosol (pH 7.4), mt-Keima shows an excitation peak at 440 nm. Following mitophagy, in acidic lysosomes (pH 4.5), the peak shifts to 586 nm [24]. The quantitative analysis of mt-Keima expressing C4da neurons revealed that the mitophagy level increased by about three-fold after vincristine treatment (Figure 2C,D), further supporting vincristine-induced mitochondrial dysfunction in C4da neurons.

Our previous study discovered that PDH inhibition protects neurons from oxidative stress in *Drosophila* models [15]. Since PDH produces H_2_O_2_ during pyruvate oxidation or mitochondrial dysfunction, inhibiting PDH can hinder ROS production in the mitochondria [14]. To assess the impact of PDH inhibition on vincristine-induced mtROS production, we aimed to knock down *Pdha1*, which encodes the PDH E1 subunit, the first component of the PDH complex [25]. When we expressed a *Pdha1* RNAi in the neuronal tissues of the flies, they developed into adults successfully, and we confirmed the down-regulation of *Pdha1* mRNA levels in the flies (Figure S1). Upon expressing *Pdha1* RNAi in C4da neurons, the flies grew into third-instar larvae, allowing us to measure the mtROS levels in *Pdha1* knocked-down C4da neurons. Although *Pdha1* RNAi did not induce any significant change in mtROS levels in control larvae, it significantly reduced the mtROS increase in vincristine-treated larvae (Figure 2A,B). Furthermore, *Pdha1* RNAi also effectively inhibited mitophagy induction in C4da neurons following vincristine treatment (Figure 2C,D), indicating that PDH inhibition can alleviate vincristine-induced mitochondrial dysfunction in C4da sensory neurons.

### 3.3. PDH Inhibition Ameliorates Thermal Hypersensitivity upon Vincristine Treatment

To further investigate the effect of PDH inhibition on vincristine-induced phenotypes in *Drosophila*, we examined thermal hypersensitivity in PDH-inhibited larvae receiving vincristine treatment. When we expressed *Pdha1* RNAi in C4da neurons, the MWL of the vincristine-treated larvae increased substantially to nearly match the level of control larvae not exposed to vincristine (Figure 3A). PDH complex activity is tightly regulated by the phosphorylation of the PDH E1 subunit [25]. Under various stresses, pyruvate dehydrogenase kinase (PDK) phosphorylates this subunit and inhibits the PDH complex, while pyruvate dehydrogenase phosphatase (PDP) dephosphorylates and reactivates it [25]. Based on this regulatory mechanism, we attempted to inhibit PDH through its regulators. Consistent with *Pdha1* knockdown, a loss-of-function mutation in *PDP*, the PDH activating gene, significantly increased the MWL during vincristine treatment (Figure 3B), and overexpression of PDK, the PDH inhibiting enzyme, in C4da neurons also reduced heat sensitivity induced by vincristine (Figure 3C). These results demonstrated that PDH inhibition alleviates thermal hypersensitivity following vincristine treatment.

### 3.4. PDH Inhibition Rescues C4da Neuron Alterations During Vincristine Treatment

When we analyzed the dendritic morphology of C4da neurons, the expression of *Pdha1* RNAi induced no meaningful change in their dendritic arborization without vincristine. However, it significantly reduced the dendritic branch points and dendritic length of C4da neurons under vincristine treatment (Figure 4A,B), further confirming that PDH inhibition can rescue peripheral neuropathy phenotypes induced by vincristine.

## 4. Discussion

In earlier studies, various models of vincristine-induced neuropathy, both in vitro and in vivo, were developed using rodents [5]. However, inconsistencies in the dosing and administration of vincristine have complicated preclinical studies involving these models [5]. Additionally, high-throughput analysis for identifying new pathophysiological mechanisms underlying vincristine-induced neuropathy was not feasible due to the challenges associated with genetic manipulation in these traditional models. Consequently, we constructed a novel VIPN model using *Drosophila*, which is well-suited for high-throughput screening and efficient genetic manipulation [10]. Our VIPN fly model demonstrated dose-dependent thermal hypersensitivity following vincristine treatment (Figure 1A,B). Higher individual and cumulative doses correlate with more severe sensory symptoms in VIPN patients [5], suggesting that our fly model can replicate the symptoms experienced by human patients. Furthermore, vincristine treatment significantly increased the number of dendrite branches and the length of C4da sensory neurons (Figure 1C,D). These dendrites innervate the epidermis and detect various stimuli, including heat [26], so alterations in the arborization of C4da neurons may lead to thermal hypersensitivity upon vincristine exposure. Moreover, *Drosophila* sensory neuron dendrites exhibit dynamic plasticity, displaying both extension and retraction events [27], similar to sensory axons found in mammalian skin [28]. Recent studies indicate that another anti-microtubule agent, paclitaxel, stabilizes terminal dendrites and inhibits terminal branch retraction, resulting in increased terminal dendrite density [19]. In the early third-instar stage, dendritic branches in C4da neurons undergo more de novo formation and extension than retraction and elimination, leading to increased dendritic length and branching. In contrast, in the late third-instar stage, branch retraction and reductions overwhelm branch formation and extension, resulting in a notable decrease in branches [29]. Although paclitaxel does not impact the arborization of C4da neurons in the early stage, it significantly suppresses dendrite retraction in the late stage [19]. These data suggest that anti-microtubule agents such as paclitaxel and vincristine negatively affect C4da neurons: they disrupt the pruning of unnecessary dendrites and heighten the neurons’ sensitivity to noxious stimuli. Consistent with this notion, when dendrite pruning in the late stage was inhibited by suppressing Notch signaling, the larvae became more sensitive to thermal stimuli [29]. These consistent findings position our model as a new platform for investigating the roles of dendrite plasticity in VIPN pathology.

Beyond its antimicrotubular activity, it has been reported that vincristine also damages mitochondria. Vincristine inhibits mitochondrial Ca^2+^ transport and disrupts intracellular Ca^2+^ homeostasis [30]. Moreover, vincristine reduces mitochondrial membrane potential and increases mtROS production in dorsal root ganglion (DRG) neuron cells [22,31]. In C4da sensory neurons, vincristine significantly elevated the mtROS level and induced mitophagy, the selective autophagy process to degrade damaged mitochondria (Figure 2). These findings suggest that vincristine-induced neuronal damage is closely linked to mitochondrial dysfunction. In our previous experiments, inhibition of PDH, the mitochondrial enzyme complex catalyzing the first step of glucose oxidation, protects dopaminergic (DA) neurons against oxidative stress in the *Drosophila* Parkinson’s disease (PD) model [15]. When we knocked down *Pdha1* in C4da neurons, the vincristine-induced mtROS levels were significantly suppressed (Figure 2), indicating that PDH is a major source of mtROS induced by vincristine treatment. In biochemical studies, PDH and a TCA cycle enzyme, alpha-ketoglutarate dehydrogenase (KGDH) account for nearly half of the total mH_2_O_2_ and exhibit production rates several times higher than complex I, the canonical source of mtROS [14]. These two enzymes are multimeric complexes composed of E1, E2, and E3 subunits. The FAD center in the E3 generates mH_2_O_2_ during forward electron transfer following pyruvate or alpha-ketoacid decarboxylation, which is the first step in the production of Acetyl-CoA or Succinyl-CoA. Additionally, PDH and KGDH can produce mH_2_O_2_ through reverse electron transfer from accumulated NADH when complex I and the electron transport chain (ETC) are impaired [14]. To check whether vincristine directly affects PDH, we measured the PDH activity in vincristine-treated third-instar larvae. In this assay, no significant difference in PDH activity was observed between control and vincristine-treated larvae (Figure S2), suggesting that vincristine induces mtROS production in PDH not through forward electron transfer from increased pyruvate oxidation but via reverse electron transfer under mitochondrial dysfunction. Further analysis revealed that PDH inhibition also alleviates vincristine-induced neuropathic phenotypes such as thermal hypersensitivity (Figure 3) and sensory dendrite alteration (Figure 4). Overall, these results indicate that mitochondrial dysfunction plays a crucial role in VIPN pathology, emphasizing PDH as a potential target for VIPN treatments.

## 5. Conclusions and Future Perspectives

In this study, we developed a *Drosophila* VIPN model that exhibited disease-related defects, including thermal hypersensitivity and alterations in sensory neuron dendrites. Further analysis revealed that vincristine stimulated mtROS production and mitophagy in the sensory neurons of our model, while inhibiting PDH effectively reduced vincristine-induced mtROS and restored sensory abnormalities. Consistent with our findings, neuronal tissues from cancer patients treated with vincristine show elevated oxidative stress damage [32]. Moreover, vincristine causes localized mtROS production and subsequent axon degeneration in iPSC-derived human neurons, and this neurotoxicity can be alleviated by mitoQ, a mitochondria-targeted antioxidant [33]. With these translational data, our results suggest that PDH inhibition represents a novel strategy to decrease oxidative stress and protect sensory neurons during vincristine chemotherapy. However, loss-of-function mutations in PDH subunit genes can cause lactic acidosis and various neurodevelopmental issues in infants [34]. Therefore, PDH inhibition would pose risks to neuro-developing infants and children, and substantial translational and clinical studies are needed to mitigate potential adverse effects and enhance the therapeutic efficacy of the PDH inhibition strategy.

## Data Availability

Data are all contained within the article.

## Supplementary Materials

The following supporting information can be downloaded at https://www.mdpi.com/article/10.3390/biomedicines13040783/s1, Figure S1: Confirmation of Pdha1 knockdown in *Drosophila*. Figure S2: PDH activity in *Drosophila* larvae is not affected by Vincristine treatment.
