# Supplementary material for: PDH Inhibition in Drosophila Ameliorates Sensory Dysfunction Induced by Vincristine Treatment in the Chemotherapy-Induced Peripheral Neuropathy Models
Source: Biomedicines. 2025 Mar 24;13(4):783. doi: 10.3390/biomedicines13040783 (PMC12025153; doi:10.3390/biomedicines13040783)
Supplement: Supplementary file 1 [file biomedicines-13-00783-s001.zip › biomedicines-3511124-supplementary.pdf]

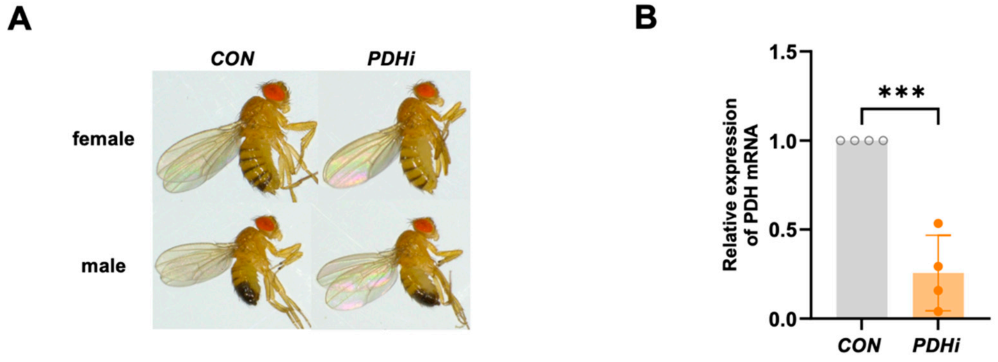

Figure S1. Confirmation of *Pdha1* knockdown in *Drosophila*. (A) Light microscopy images and (B) *Pdha1* mRNA levels of control (*CON*) and *Pdha1*-knockdown (*PDHi*) flies. (n = 4 per group). Significance was determined by Student's t-test (***, P<0.001). An error bar indicates the mean ± SD.

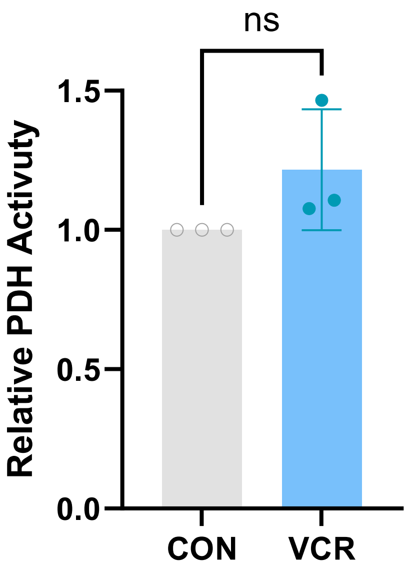

Figure S2. PDH activity in *Drosophila* larvae is not affected by Vincristine treatment. Relative PDH activity of 3rd instar larvae treated with vehicle (CON) or 100 μM vincristine (VCR) for 48 h (n = 3 per group). Significance was determined using Student's t-test (ns, not significant). Error bars represent mean ± SD.
